# High Prevalence of Diarrhoegenic Intestinal Parasite Infections among Non-ART HIV Patients in Fitche Hospital, Ethiopia

**DOI:** 10.1371/journal.pone.0072634

**Published:** 2013-08-26

**Authors:** Haileeyesus Adamu, Teklu Wegayehu, Beyene Petros

**Affiliations:** 1 Department of Biology, Addis Ababa University, Addis Ababa, Ethiopia; 2 Tropical and Infectious Diseases, College of Health Sciences, Addis Ababa University, Addis Ababa, Ethiopia; 3 Department of Microbial, Cellular and Molecular Biology, College of Natural Sciences, Addis Ababa University, Addis Ababa, Ethiopia; Tulane University School of Public Health and Tropical Medicine, United States of America

## Abstract

**Background:**

HIV infection has been modifying both the epidemiology and outcome of parasite infections. Hence, this study was undertaken to determine the prevalence of *Cryptosporidium* and other intestinal parasite infections among HIV positives with and without Antiretroviral Treatment(ART) and its association with CD4+ T-cell count.

**Methods:**

A cross-sectional study was conducted at Fitche hospital focusing on HIV positives who came to hospital for follow-ups. A total of 378 HIV positive persons with and without ART participated in the study. Data on socio-demographic factors and diarrhoea status were obtained by interviewing all 214 with ART and 164 without ART. Stool samples were collected from all patients and examined for intestinal parasites using direct, formol-ether and modified acid-fast staining techniques.

**Results:**

The prevalence of intestinal parasite infections in this study was significantly higher among HIV positive persons not on ART. Specifically, the rate of infection with *Cryptosporidium* species, *Blastocystis* spp., *Giardia lamblia*, and *Entamoeba histolytica/E. dispar* were higher, particularly in those with CD4+ T-cell counts less than 200 cells/µL. Fifty seven percent of the study participants were on ART. Out of these 164/378 (43%) of the non-ART study participants were infected with at least one intestinal parasite species. Significant association was observed between lower CD4+ T-cell count (<200 cells/µL) and the prevalence of *Cryptosporidium* spp. and *Blastocystis* spp. The two parasites were significantly more prevalent in HIV positive non-ART patients.

**Conclusion:**

HIV infection increased the risk of having *Cryptosporidium* and other intestinal parasites and diarrhoea. Therefore, raising HIV positive’s immune status and screening for intestinal parasites is important. This study showed that patients who are taking ART had a lower prevalence of diarrhoea causing parasites and *Cryptosporidium* suggesting that ART through improvement of immune status of the patients may have contributed to controlling diarrhoea-causing parasites in HIV positive patients.

## Introduction

Intestinal parasites are endemic in many regions of the world where Human Immunodeficiency Virus/Acquired Immunodeficiency syndrome (HIV/AIDS) is also prevalent. In Sub-Saharan Africa, the prevalence of intestinal parasite infections is high and the largest burden of AIDS cases exists [1]. Even though AIDS remains a global pandemic, Ethiopia is one of the highly affected Sub-Saharan African countries.

It is estimated that as much as 60% of the world’s population is infected with intestinal parasites, which may play a role in morbidity due to intestinal parasite infections [2]. Intestinal parasite infections are among the most common infections worldwide. It is estimated that some 3.5 billion people are affected, and 450 million are sick because of these parasite infections [3]. Reports indicate that diarrhoea occurs in 30–60% of AIDS patients in developed countries, whereas it reaches up to 90% in developing countries [3]. The rate of infection is remarkably high in Sub-Saharan Africa, where the majority of HIV and AIDS cases are concentrated [1]. The incidence of intestinal parasite infections reaches up to 95% in HIV positive persons in developing countries. These infections are caused both by protozoa and helminths and the main clinical manifestation of the disease caused by them is diarrhoea [4].

Studies showed the existence of interaction between HIV and intestinal parasite infections in co-infected persons. Intestinal parasite infections particularly helminths cause chronic immune activation [5], in addition to skewing the immune response towards T helper-2 immune responses [6]. Thus, chronic immune activation is suggested as one factor that adversely influences epidemics of HIV/AIDS in Africa [7]. On the other hand, HIV infection has increased the severity of the consequences of parasite infection. With the emergence of AIDS, epidemiology and outcome of diseases caused by parasites were significantly modified [8]. However, the effect of HIV on some parasites is not clearly understood.

Overall, either backed by HIV or independently, intestinal parasite infections have continued to be major cause of morbidity and mortality in humans [9]. Intestinal parasite infections frequently cause severe diarrhoea, which often is responsible for the severity of the disease, and may sometimes lead to death. The progressive decline of mucosal immunologic defence mechanisms predisposes patients to intermediate or late gastrointestinal manifestations such as diarrhoea [10]. In the late stages of AIDS, the alterations of non-specific defence mechanisms, production of IgA antibodies and local cellular immune responses also progress, thus increasing the susceptibility to various intestinal parasites, such as *Cryptosporidium* species, *Isospora belli,* and Microsporidia [11], [12].

Ethiopia is among the sub-Saharan countries with overlapping high rate of HIV and parasite infections. There have been few studies reported on epidemiology of intestinal parasites on HIV positive people. The present study is aimed to determine the prevalence of intestinal parasites in HIV positive persons with and without Antiretroviral Treatment (ART) and their association with diarrhoea and immune status.

## Materials and Methods

### The Study Area

The study was carried out in Fitche hospital, which is the main ART centre in the area, 114 km from Addis Ababa, Ethiopia from February 2010 to December 2010. The hospital uses two approaches for HIV/AIDS counselling and testing: client-initiated to serve people seeking to know their HIV status and provider-initiated to enable health care provider offer specific medical services. People who test HIV- positive at the hospital and referred from other health institutions are attached to the ART clinic for clinical and laboratory investigations to monitor their immune status and viral load. Immunological assessment using CD4+ T-cell count at enrolment and follow-up visits is used to identify those who are eligible for ART.

### The Study Population

The study population consisted of HIV-positive individuals, who gave blood for CD4+ T-cell count at their first enrolment to monitor disease status at the ART clinic during the study period. Clients tested HIV-negative at the hospital in the same period and patients receiving anti-parasite treatment were excluded from the study. Socio-demographic and clinical information including diarrhoea and medication histories were obtained from the study participants by interview and their CD4+ T-cell counts were obtained from their medical records at the ART centre. CD4+ T-cell counts taken from their clinical history and considered only when it is done at the time of or very close to stool sample collection (within a week period). In total, 378 HIV positive people (214 with ART and 164 without ART) were included in the present study.

### Stool Collection and Processing

A single fresh stool sample was collected from consenting study participants (n = 378). The physicians or principal investigator filled the questionnaires in this study during sample collection. A portion of the stool was preserved in 10% formalin in a proportion of 10 g of stool in 3 ml of formalin.

### Direct Microscopy (Wet Mount)

A direct wet mount of stool with normal saline (0.85% NaCl solution) was prepared at hospital; and examined for the presence of motile intestinal parasites and trophozoite under light microscope (400×magnification). Lugol’s iodine staining was used to detect cysts of intestinal parasites.

### Formalin-Ether Concentration

Using an applicator stick, about ∼5 g of preserved stool sample was placed in a clean 15 ml conical centrifuge tube containing 7 ml formalin. The sample was dissolved and mixed thoroughly with applicator stick. The resulting suspension was filtered through a sieve (cotton gauze) into a beaker and the filtrate poured back into the same tube. The debris trapped on the sieve was discarded. After adding 3 ml of diethyl ether to the mixture and hand shaken, the content was centrifuged at 2000 rpm for 3 minutes. The supernatant was poured away and the tube was placed in its rack. The sediments were stained with Iodine, put on slide and covered with cover slip. The entire area under the cover slip was examined using 400×objective magnification [13]. Microscopic examinations were done independently by experienced clinical laboratory technicians; the determination & verification was done by the principal investigator.

### Modified Zeihl-Neelsen Method

Modified Zeihl-Neelsen staining; based on direct and concentration methods, for detection of opportunistic coccidial intestinal parasite oocysts of *Cryptosporidium* spp., *Isospora belli*, *Cyclospora cayetanensis* was done. Fresh faecal samples were collected and thin smears were prepared, air-dried, fixed with methanol for 5 minutes in the field and stained by Zeihl-Neelsen technique at Biomedical Sciences Laboratory Department Biology, Addis Ababa University. The same procedures were used for smears prepared after concentration. Smears were prepared from the concentrated stool samples and were stained as described by Adegbola [14] with some modifications. In this technique, the slides were stained with carbol-fuchsine for 30 minutes and then after, were washed with tap water. The slides were decolorized in 1% HCl acid-alcohol for 1 minute and were counter stained with 1% methlyene blue for another 1 minute. Finally, the stained smears were microscopically observed using 1000×magnification [15].

### Data Analysis

SPSS Version-20 software was used for registering data and analysis. Mean, range and percentage were used to describe different characteristics of study subjects as appropriate. Chi square and Fisher Exact tests were used to determine p-value. A given statistical test was reported significant if it resulted in a p-value<0.05.

### Ethical Consideration

This study was conducted with the approval of the Ethical Review Committee of Research, Department of Biology, Addis Ababa University. Informed written consent was taken from each study participant. Participants were also informed that they are free to withdraw consent at any time and their records and specimen will be examined by authorized persons, and all personal information on them will be treated confidential. Clinicians managed those participants positive for intestinal parasites.

## Results

The majority of the study participants were urban residents (80.7%) and others were from the rural vicinities of Fitche town. Most of the study participants (76.2%) were in the age range 20–40 years old, 9.3% were less than 20 years old age, and 14.6% were above 40-year old age. Of the total study participants, on ART were 56.6%. The mean age of participants with ART was 30.2 years (range = 17–67 years) compared with mean age of 27.2 years (range 14–71 years) in non-ART people.

Based on parasitological examination of the stool specimens, 13 species of intestinal parasites were detected in 63.5% (234/378) HIV positive patients. The detected parasites included protozoa and helminths: *Cryptosporidium* species, *Blastocystis spp., Isospora belli*, *Giardia lamblia*, *Entamoeba histolytica/E. dispar*, *Hymnolepis nana, Enterobius vermicularis, Schistosoma mansoni, Taenia spp., Trichuris trichiura, Ascaris lumbricoids,* hookworms, and *Strongyloides stercoralis*. The most frequently detected parasites were *Cryptosporidium* species in 32 (8.5%) patients and *Blastocystis* spp. in 24 (6.3%) patients (Table 1).

**Table 1:** Prevalence of intestinal parasites among HIV positive patients with and without ART in Fitche Hospital, Ethiopia, 2010.

Parasites	HIV Positives with ART(n = 214) Number (%)	HIV Positives without ART (n = 164)Number (%)
**Protozoa**		
*Cryptosporidium* spp.	0	32 (19.5)
*Blastocystis* spp.	3 (1.4)	21 (12.8)
*Isospora belli*	0	6 (3.6)
*Giardia lamblia*	8 (3.7)	7 (4.3)
*E. histolytica/E. dispar*	7 (3.3)	9 (5.5)
**Helminths**		
*Hymnolepis nana*	1 (0.5)	5 (3)
*Enterobius vermicularis*	4 (1.9)	3 (1.8)
*Schistosoma mansoni*	2 (0.9)	3 (1.8)
*Taenia* spp.	5 (2.3)	5 (3)
*Trichuris trichiura*	5 (2.3)	3 (1.8)
*Ascaris lumbricoids*	7 (3.3)	4 (2.4)
Hookworms	0	2 (1.2)
*Strongyloides stercoralis*	1 (0.5)	9 (5.5)
Total	118 (55.1)	116 (70.7)

Among HIV positive patients without ART, 70.7% (116/164) were infected with intestinal parasites compared with 55.1% (118/214) of HIV positive patients with ART. *Cryptosporidium* spp., *I. belli* and hookworms occurred exclusively among HIV positive persons without ART. Moreover, almost all cases (except one case of each) of *S. stercoralis* and *H. nana* infection occurred among HIV positive persons without ART. The association of these intestinal parasite infections in HIV positive persons without ART was significant. Infection of *G. lamblia*, and *E. histolytica/E. dispar* were higher in HIV positive persons without ART but was not significantly associated (Table 1).

HIV positive patients without ART reported increased rate of chronic diarrhoea (Table 2) whereby HIV positive patients without ART were more than two times likely to have chronic diarrhoea relative to HIV positive patients with ART. Acute diarrhoea was reported at a higher rate among HIV positive patients without ART (p = 0.04) where 43% (50/116) of HIV positive patients without ART, but infected with parasites, reported to have acute diarrhoea. This rate was significantly higher compared to HIV positive patients with ART and with parasite infections (p = 0.03). In terms of causing diarrhoea, ART and parasite infections had significant interaction (Table 2). HIV and parasite co-infection had reported to have higher risk of any type of diarrhoea.

**Table 2:** Association of diarrhoea among HIV positive patients with and without ART stratified by parasite infection in Fitche Hospital, Ethiopia, 2010.

Type of Diarrhoea	HIV Positive with ART (n = 214) Number (%)		HIV Positive without ART (n = 164) Number (%)		Total		p - value
	Parasite Positive	Parasite Negative	Parasite Positive	ParasiteNegative	With ART	Without ART	
Acute	25 (21.2)	18 (18.7)	50 (43.1)	3 (6.2)	43 (20.1)	47 (42.6)	0.04
Chronic	26 (22)	28 (29.2)	35 (30.2)	7 (14.6)	54 (25.2)	19 (11.6)	0.03
No diarrhoea	67 (56.8)	50 (52.1)	31 (26.7)	38 (79.2)	117 (54.7)	98 (59.8)	Referent
Total	118 (55.1)	96 (44.9)	116 (70.3)	48 (29.7)	214 (56.6)	164 (43.3)	

Remark: the percent values were calculated as the total within that group.

Statistically significant at p<0.05.

*E. histolytica/E. dispar* was more likely detected among HIV positive patients without ART who reported acute diarrhoea compared with those with chronic diarrhoea. However, *G. lamblia* was frequently detected in those with acute and chronic diarrhoea.

The prevalence of parasite infection was increased with decreasing CD4+ T-cell count among HIV positive patients without ART (Table 3). The highest infection prevalence was at CD4+ T-cell counts less than 200 cells/µL and it was about five times higher than individuals having CD4+ T-cell counts greater than 500 cells/µL. HIV positive patients without ART with CD4+ T-cell counts less than 200 cells/µL had reported higher risk of having diarrhoea independent of parasite infection compared with those having 500 cells/µL and above.

**Table 3:** Prevalence of parasite species in different groups of CD4+ T-cell counts in Fitche Hospital, Ethiopia, 2010.

CD4+ count	Type of Parasite						p - value
	*Cryptosporidium*spp. n (%)	*I. belli* n (%)	*Blastocystis* spp.n (%)	*S. stercoralis*n (%)	Any parasiten (%)	Total positivesn (%)	
<200 cells/µl	29 (29.6)	5 (5.1)	20 (20.4)	6 (6.1)	38 (38.8)	98 (64.5)	0.03
200–499 cells/µl	2 (5.5)	1 (2.8)	3 (8.3)	2 (5.6)	28 (77.8)	36 (23.7)	0.02
≥500 cells/µl	1 (5.5)	0	1 (5.5)	2 (11.1)	14 (77.8)	18 (11.8)	Nd
Total	32 (21)	6 (3.9)	24 (15.8)	10 (6.6)	80 (52.6)	152	

Statistically significant at p<0.05: Nd: not determined.

## Discussion

The study has shown a similar prevalence (63.5%) of intestinal parasite infections as reported (39.8%) earlier among individuals with and without HIV/AIDS in south western Ethiopia [16]. This indicates the similarity in the hygienic and health service conditions between the two study locations of Ethiopia. The opportunistic parasites, *I. belli, Cryptosporidium* spp. and *Blastocystis spp., which* were detected previously from AIDS patients and HIV positive persons without ART, in Addis Ababa [17] [18] and elsewhere [11], were also detected significantly more times in non-ART diarrhoeic HIV patients in the present study. The concurrence of findings indicates the relevance of ART in preventing opportunistic parasites from inducing diarrhoea in HIV infected persons.

As diarrhoea is an important gastrointestinal syndrome in HIV/AIDS patients, a comparison conducted between the associated intestinal parasites in diarrhoeic and non-diarrhoeic patients. Among the faecal samples for parasites, the diarrhoeic states were closely related with the presence of parasites in the stool samples. This association is in agreement with other studies showing that only 20% of the diarrhoeic patients with AIDS presented an obscure etiology, whereas in more than 50% (of patients) parasites were diagnosed [19].

The significant association between parasite positivity and diarrhoea was true for *Cryptosporidium* spp., *G. lamblia*, *I. belli, E. histolytica/E. dispar* and *Blastocystis spp.* infections. Previous studies in Ethiopia have also shown among patients infected with HIV, an infection rate of 5% and 8% for *I. belli* and *Cryptosporidium* spp., respectively [11] [15] [17] [18].

Although the role of *I. belli* as an opportunistic infection in persons with AIDS is prominent [19] and it appears to be common in Ethiopia, there are only few published reports so far [11] [16] [20]. The actual rate of this infection in immunocompetent individuals is likely to be underestimated because of the healthy appearance of asymptomatic carriers that shed oocysts and treatment with cotrimoxazole and other antibiotics for other infections may confer some protection against this parasite in AIDS patients [21]. Although the prevalence of cryptosporidiosis in humans is relatively high in Ethiopia, there is only one report that confirmed the species responsible to be *C. parvum* (99%) and *C. hominis* (1%) [22].

This study showed that intestinal parasite infections are common among HIV positive patients without ART and that there exists a relationship between the type of parasite and CD4+ T-cell count. The prevalence of opportunistic protozoan parasites tended to be higher in HIV positive patients with lower CD4+ T-cell count. This finding is similar to reports from Ethiopia [18] and Cameroon [23]. The prevalence of intestinal parasites reported in this study (40.2%) is lower than that reported (52.6%) from a study at Jimma University Teaching Hospital in Ethiopia [20]. This variation could be owing to environmental hygiene differences, economic, educational status of the study subjects and climatic conditions.

The prevalence rates of *Cryptosporidium* spp., *I. belli* and *Blastocystis. spp.* were higher in comparison to the rate reported by Awole and his associates [16] from South Western Ethiopia. It is known that geographical location (altitudinal difference between the two locations; climatic conditions) and levels of general hygiene play a role in the distribution of parasites. Although, *Blastocystis. spp.* is believed to be non-pathogenic by many, the present finding is an additional evidence to its involvement in diarrhoea, especially in immunocompromised HIV/AIDS patients [24].

The overall prevalence of intestinal parasites, which was significantly higher in HIV positive patients who did not start ART and hence had lower CD4+ T-cell count, is an indication that several parasitic infections are associated with immunosuppression, which may enhance parasite establishment and may thus increase parasite load [25]. This in agreement with the report of Mekonnen [26] who showed a higher prevalence of intestinal helminth infections among HIV infected individuals at the time of initiation of ART in patients whose CD4+ T cell count is less than 200 cells/µL than among non-ART HIV-positives whose CD4+ T cell count is more than 200 cells/µL.

The prevalence of opportunistic parasites, *Cryptosporidium* spp. and *I. belli,* were significantly higher in HIV/AIDS patients who were not enrolled in ART when compared with those enrolled in ART and with CD4+ T-cell count >200 cells/µL. This can be explained by the fact that there will be low opportunity for these parasites to proliferate and cause diarrhoea as the patient’s CD4+T cell count increases. Although studies assessing reduction in the incidence of cryptosporidiosis is lacking, diarrhoea due to cryptosporidia are known to resolve spontaneously with immune restoration among HIV/AIDS patients on ART [27], [28], [29].

It is possible that some parasites were not detected in this study because not all techniques, such as the water-ether sedimentation method for *Microsporidia* or adhesive tape or anal swab for *Entrobium vermicularies* and the Baerman technique or culture for *S. strongyloides, etc* were used. Therefore, the prevalence of intestinal parasites among the study participants may have been underestimated.

From this study, we can conclude that ART will reduce diarrhoea prevalence caused by the opportunistic protozoa - *Cryptosporidium* spp., *I. belli* and *Blastocystis* spp.- infections in HIV/AIDS patients. This data supports the value of standard faecal examinations in HIV positive individuals, since these examinations can be easily performed with low cost allowing initiation of affordable therapies. Overall, more effort is needed to provide easy access to HIV care services by expanding the availability of ART and constantly reinforcing patient adherence to antimicrobial therapies and prophylaxis, especially for immunocompromised and HIV/AIDS patients.

Based on this study we recommend: public health measures that emphasize the importance of environmental and personal hygiene; monitoring the quality of drinking water; more education and training to health practitioners and laboratory technicians about emerging diarrhoea-causing parasites that could compromise the health of people living with HIV/AIDS; future efforts on the opportunistic protozoan intestinal parasites should determine their zoonotic potential by determining their prevalence in domestic animals by using genotyping techniques. Since microscopic determinations are poorly sensitive and inadequately discriminate pathogenic from non-pathogenic parasite species, a well-designed molecular study is recommended to determine the true prevalence of opportunistic parasite infections in HIV positive persons in Ethiopia.
